# Evaluation of residual stress and texture in isotope based Mg^11^B_2_ superconductor using neutron diffraction

**DOI:** 10.1039/c8ra05906c

**Published:** 2018-11-26

**Authors:** Hyunseock Jie, Wenbin Qiu, Daniel Gajda, Jeonghun Kim, Valiyaparambil Abdulsalam Anvar, Arend Nijhuis, Yoshio Bando, Yusuke Yamauchi, Vladimir Luzin, Md. Shahriar A. Hossain

**Affiliations:** Australian Institute for Innovative Materials (AIIM), University of Wollongong Squires Way North Wollongong NSW 2500 Australia shahriar@uow.edu.au; Australian Nuclear Science & Technology Organisation (ANSTO) Lucas Heights NSW 2232 Australia vll@ansto.gov.au; Institute of Low Temperature and Structure Research Polish Academy of Sciences ul. Okólna 2 50-422 Wrocław Poland; School of Chemical Engineering, Australian Institute for Bioengineering and Nanotechnology (AIBN), The University of Queensland Brisbane QLD 4072 Australia y.yamauchi@uq.edu.au; The University of Twente, Faculty of Science & Technology 7522 NB Enschede Netherlands; International Center for Materials Nanoarchitectonics (WPI-MANA), National Institute for Materials Science (NIMS) 1-1 Namiki Tsukuba Ibaraki 305-0044 Japan; Department of Plant & Environmental New Resources, Kyung Hee University 1732 Deogyeong-daero, Giheung-gu Yongin-si Gyeonggi-do 446-701 South Korea; School of Mechanical and Mining Engineering, The University of Queensland Brisbane QLD 4072 Australia

## Abstract

Magnesium diboride (MgB_2_) superconducting wires have demonstrated commercial potential to replace niobium–titanium (NbTi) in terms of comparable critical current density. Its higher critical temperature makes MgB_2_ wire suitable for liquid-helium-free operation. We recently reported boron-11 isotope-based low-activation Mg^11^B_2_ superconducting wire with decent critical current density appropriate for low-cost superconducting fusion magnets. In this study, we have mainly focused on the neutron diffraction technique to measure the residual stress in Mg^11^B_2_ superconducting wire for the first time. The residual stress state was given qualitative and quantitative interpretation in terms of micro- and macrostress generation mechanisms based on the isotropic model confirmed by neutron texture measurements. The relationship between the stress/strain state in the wire and the transport critical current density is also discussed. This investigation could pave the way to further enhancement of the critical current density of low-activation Mg^11^B_2_ superconducting wires suitable for next-generation fusion grade magnets.

## Abstract 2

Magnesium diboride (MgB_2_) superconducting wires have demonstrated commercial potential to replace niobium–titanium (NbTi) in terms of comparable critical current density.

## Graphical abstract

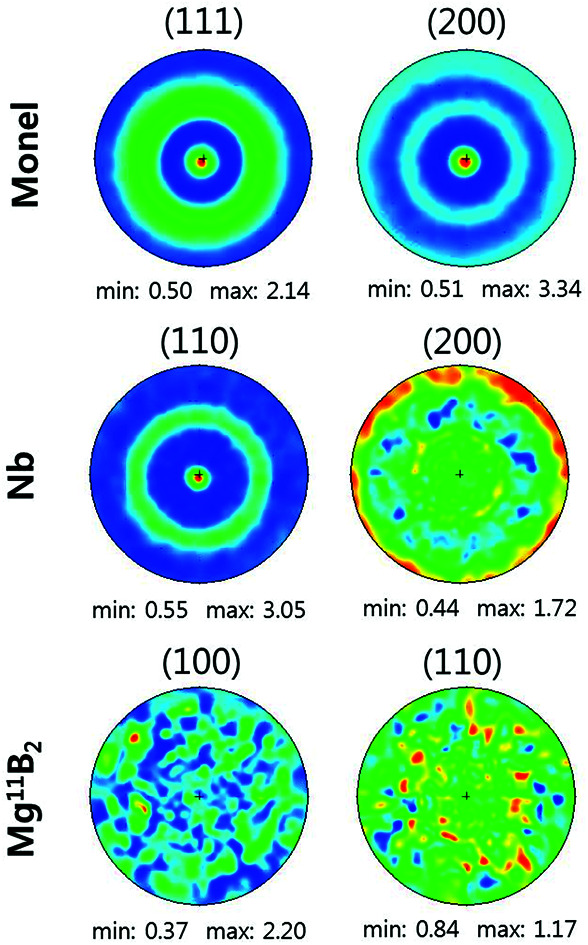

## Introduction

While the current workhorse superconductors for the International Thermonuclear Experimental Reactor (ITER) are low-temperature NbTi and Nb_3_Sn superconductors,^1–3^ MgB_2_ shows electromagnetic performance superior to that of NbTi: it has lower induced radioactivity,^4,5^ higher efficiency of the cryogenic reactor system,^6–8^ and a much higher transition temperature (*T*_c_).^9,10^ Furthermore, the field performance, in terms of its transport critical current density (*J*_c_) and upper critical field (*B*_c2_), is close to that of NbTi superconductor.^11–13^ Thus, MgB_2_ is possibly a viable candidate to replace NbTi superconductors in the poloidal field (PF) coils and correction coils (CC) for the next-generation fusion reactors. Based on the analysis reported by Devred *et al.* and Hossain *et al.* of the conductor development and performance criteria for the ITER project, the critical current capacity of MgB_2_ cables clearly fulfils the requirements for use in the PF and CC magnets, even at 20 K, in the ITER fusion reactor.^13,14^ The use of conduction-cooled low-cost MgB_2_ at 20 K in PF and CC magnets to replace NbTi will make the next generation fusion reactor much more cost effective. MgB_2_ wire filament is brittle after the heat treatment, and given the strain limit criterion of 0.2% for the magnet design, the maximum strain limit is well below 0.2% to provide a factor of two safety margin.^15^ Despite the prospects for the use of MgB_2_ as a fusion reactor superconducting material, many technological issues need to be resolved, and the current work aims to report the progress in this direction.

The critical point in reactor application is the use of boron-11 isotope enriched powder for the fabrication of the MgB_2_ superconductor. Natural boron has 19.78 wt% boron-10 (^10^B) and 80.22 wt% boron-11 (^11^B).^16–18^^10^B is well known as a neutron absorption material with a large nuclear reaction cross-section, leading to transformation into ^7^Li and He *via* the (n, *α*) reaction.^19–21^ In contrast, ^11^B is stable in the presence of neutron irradiation without an (n, *α*) reaction and can reduce nuclear heating.^22,23^ Therefore, ^11^B isotope based Mg^11^B_2_ superconductor is the most desirable, if not absolutely necessary, material for Tokamak type magnets in fusion reactors.

In recent ITER superconducting cable performance tests, damage to the superconducting filaments has been recognised as a significant issue.^24^ It was demonstrated that the superconducting filaments in the cables are easily damaged when exposed to temperature and electromagnetic cycling, simulating ITER operational regimes, and that this microscale damage has a detrimental effect on superconducting properties. The root cause of the microscale damage is associated with electromagnetic (Lorentz) forces as well as thermally generated stresses due to cooling to cryogenic temperatures and residual stresses generated during the production process.^25–28^ Therefore, it is essential to assess and, if possible, to control the stress and strain state of the filaments, both when it originates from the manufacturing process and when it occurs due to the operating conditions. This knowledge can be used to predict and, ideally, eliminate possible damage to the superconducting filaments. In this respect, the residual stress is not only a partial cause of the damage, but also a quantity that can be studied to assess the degree of microscale damage. In case of development of microscale damage, the residual stresses become relaxed to a certain degree, and this effect can be studied experimentally.

Knowledge of the residual stresses is also important for understanding the effects of applied stress/strain on the superconducting properties, *i.e.* the critical current (*I*_c_), which have been experimentally observed multiple times in MgB_2_ superconducting systems.^29–32^ The residual stresses were measured successfully on several occasions for Nb_3_Sn using neutron diffraction,^33,34^ and this technique proved to be the most suitable for the powder-in-tube system due to its ability to penetrate through the sheath material. There are no published results on measurements of the residual stress in MgB_2_ wires, however, presumably due to the fact that manufacturing ^11^B isotope based Mg^11^B_2_ wires is a prerequisite for such neutron measurements. Nevertheless, it is conceptually clear that, depending on the sign and magnitude of the residual stress, the combined effect of the residual and applied stress/strain can be different.

From this point of view, understanding the stress/strain behaviour of the Mg^11^B_2_ wires and coils for the magnet system of a fusion reactor is a critical issue in terms of current-carrying capability. Direct stress/strain measurements on the Mg^11^B_2_ filaments in the wire are difficult, because the Mg^11^B_2_ filamentary region, for practical use, is covered with a Monel (Ni–Cu alloy) sheath and Nb barrier. A high penetration depth of radiation, such as in the form of neutrons or high-energy synchrotron X-rays, is required to measure residual stress and texture on the superconducting wire.^35^

In this report, we used neutron diffraction for a full quantitative residual stress analysis of the constituents in ^11^B isotope based Mg^11^B_2_ wires (Mg^11^B_2_), in correlation with the fabrication conditions and the transport critical current density (*J*_c_), for the first time. This assessment is the first step on the way to optimising the properties and manufacturing conditions for Mg^11^B_2_ superconductor intended for magnets in fusion reactors, with the possibility of mitigating unwanted stress and strain inside the wire filaments.

## Experimental details

The wire samples were prepared by using the conventional *in situ* powder-in-tube (PIT) method. The ^11^B low crystalline powder (from Pavezyum Kimya, Turkey), which consists of amorphous and crystalline components, was sintered by the Moissan method^36^ with 840 nm particle size and isotopic purity of 99.25 ± 0.01% of ^11^B. Magnesium powder (100–200 mesh, 99% purity), a niobium barrier, and a Monel (Ni–Cu alloy) sheath tube were also used for the production of the Mg^11^B_2_ wire. This particular ^11^B powder was chosen from a selection of several candidates on the basis of precursor powder and wire product characterisation (*e.g.* the isotopic purity reported above was determined by means of neutron transmission experiments and accelerator mass-spectrometry), and a study giving the details will be published separately elsewhere. The tube was swaged and drawn to an outer diameter of 1.08 mm, and then the wires were subjected to heat treatment at 700 °C, 750 °C, and 800 °C for 1 hour (ramp rate of 5 °C min^−1^) under a high purity argon gas atmosphere. Scanning electron microscopy (SEM, JEOL JSM-6490LV) and X-ray diffraction (XRD, GBC-MMA) were employed to observe the microstructure and the phase composition using sectioned wires. The core, Mg^11^B_2_ based ceramic, was extracted from the Nb-Monel sheath for the X-ray diffraction. The volume fractions of ^11^B-rich phase, Mg, and MgO for Mg^11^B_2_ were obtained using the MAUD program based on the X-ray diffraction.^37–41^

For neutron experiments, the individual Mg^11^B_2_ wires were cut into pieces ∼5 mm in length and bunched together to form bulk samples with approximate dimensions of 5 × 5 × 5 mm^3^. Measurements of residual stress were performed on the niobium, Mg^11^B_2_, and Monel phases. The measurements of residual stress on the Mg^11^B_2_ wires were carried out using the KOWARI neutron diffractometer^42^ at the Open Pool Australian Lightwater (OPAL) research reactor at the Australian Nuclear Science and Technology Organization (ANSTO). The Mg^11^B_2_ phase was measured in a 90° geometry using the wavelength *λ* = 1.5 Å for the Mg^11^B_2_ (211) reflection and gauge volume size of 4 × 4 × 4 mm^3^. Two principal directions, transverse and axial, were measured with constant rotation of the samples around their axis for better averaging.

A specially prepared pure Mg^11^B_2_ cylindrical pellet sample (5 mm diameter, 3 mm height) was used to determine the unstressed lattice spacing, *d*_0_. For the production of this pellet, a high temperature (HT) 800 °C thermal regime was used to produce a uniform (no Monel sheath, no Nb barrier) and high purity sample to ensure the absence of macro- and microstresses.

The stress (*σ*) was calculated for the measured transverse and axial strains, *ε*_t_ = (*d*_t_ − *d*_0_)/*d*_0_ and *ε*_a_ = (*d*_a_ − *d*_0_)/*d*_0_, respectively, of the Mg^11^B_2_ (211) reflections in the corresponding directions using the (*hkl*)-dependent Young's modulus (*E*) and Poisson's ratio (*ν*) calculated from the single crystal elastic constants in the isotropic approximation, *E* (211) = 316.2 GPa, and *ν* (211) = 0.17. The two principal stress components, transverse and axial, were computed accordingly to the following relationship 
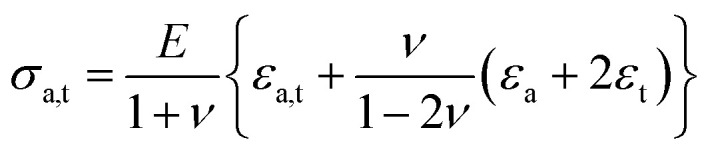
 adapted for the case of the cylindrical symmetry stress state from the general Hooke's law.^43,44^ The cylindrical symmetry of the stress state reflects and inherits the cylindrical symmetry of the wire drawing process with only two distinct directions, axial and transverse.

To further study aspects of the anisotropic stress state, neutron texture measurements were performed on the wires, including three phases, Mg^11^B_2_, Monel sheath, and Nb barrier (only for the sample sintered at 700 °C, since the other samples were essentially identical). Several representative pole figures were collected to judge the crystallographic isotropy/anisotropy using the same KOWARI diffractometer. We consider that the effect of crystallographic texture,^45^ which requires experimental determination, is three fold. First, it determines the anisotropy of the elastic and thermal properties (*e.g.* Young's modulus and the coefficient of thermal expansion), which is important for proper stress calculation procedures, as well as for stress evaluation if a finite element method (FEM) simulation is to be done. Second, if some crystallographic preferred orientation is found, it can shed light on the mechanism of MgB_2_ phase formation and growth in the sintering process. Third, for the polycrystalline layered superconductors, *e.g.* yttrium barium copper oxide (YBCO), with extremely high anisotropy of the critical current, the effect of texture is so high that the current can be practically destroyed due to unfavourable crystallographic alignment of the grains.^46^ Although the single crystal anisotropy of MgB_2_ is much less pronounced, control of the degree of preferred orientation is required.

## Results and discussion

**Fig. 1:**
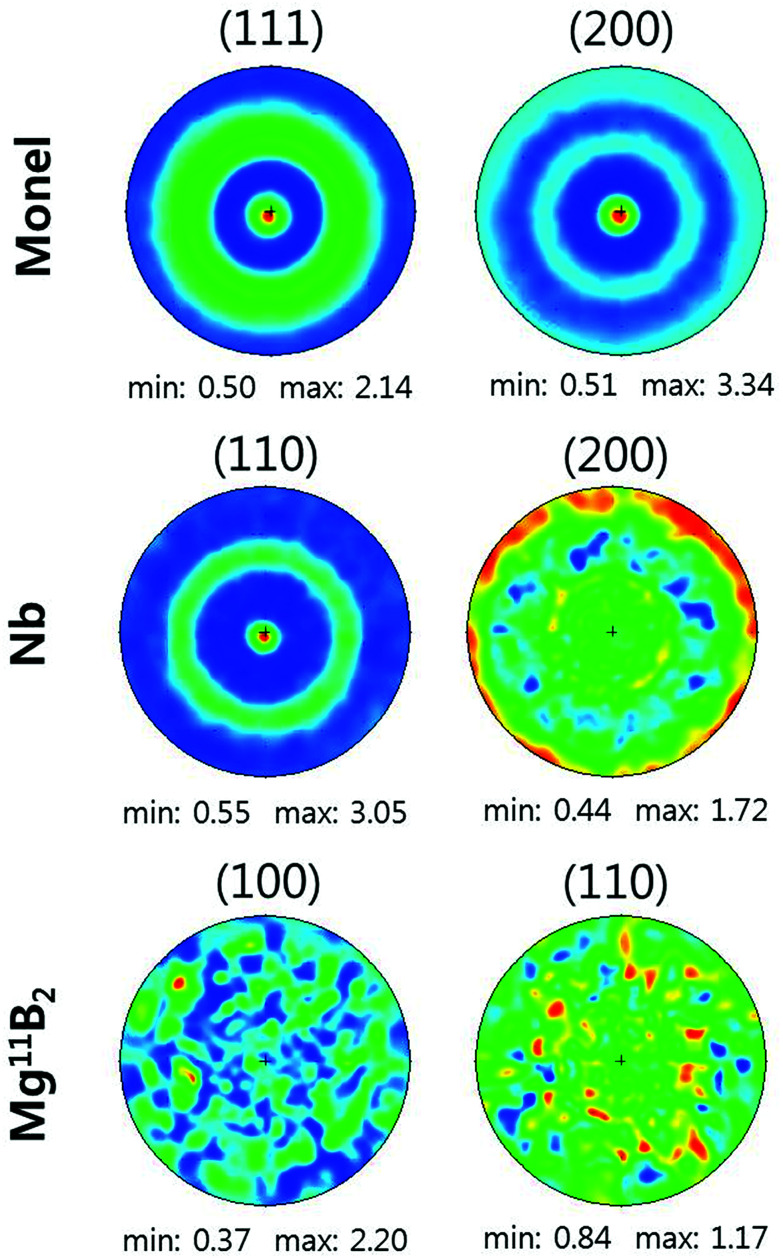
Pole figures of the phases in the Mg^11^B_2_ wire heat-treated at 700 °C.

The crystallographic anisotropy was quantified by neutron texture analysis, and the results are shown in Fig. 1 as a set of representative pole figures for the three materials used in the wire, Monel, Nb, and Mg^11^B_2_.^45^ While there is strong anisotropy in the Monel-Nb sheath due to tensile plastic deformation during the swaging process, the crystal orientation of Mg^11^B_2_ has a random distribution (with only statistical oscillations visible in the pole figures of Mg^11^B_2_, while there is no pattern with preferred orientation). Thus Mg^11^B_2_ phase is crystallographically isotropic (with no preferred crystal orientation), and therefore, the elastic properties, which are important for the stress analysis of the system, have no anisotropy related to the crystalline preferred orientation. This does not eliminate the possibility of elastic anisotropy due to other micromechanical factors, however, *e.g.* microcracking determined, for example, by the deformation process. The results of the texture analysis are to be used for macrostress calculations in the elastically anisotropic model of the sheath material and its interaction with the Mg^11^B_2_ interior, which is isotropic. The experimentally determined isotropy of the Mg^11^B_2_ interior is used here for model microstress calculations within the isotropic approximation.

**Fig. 2:**
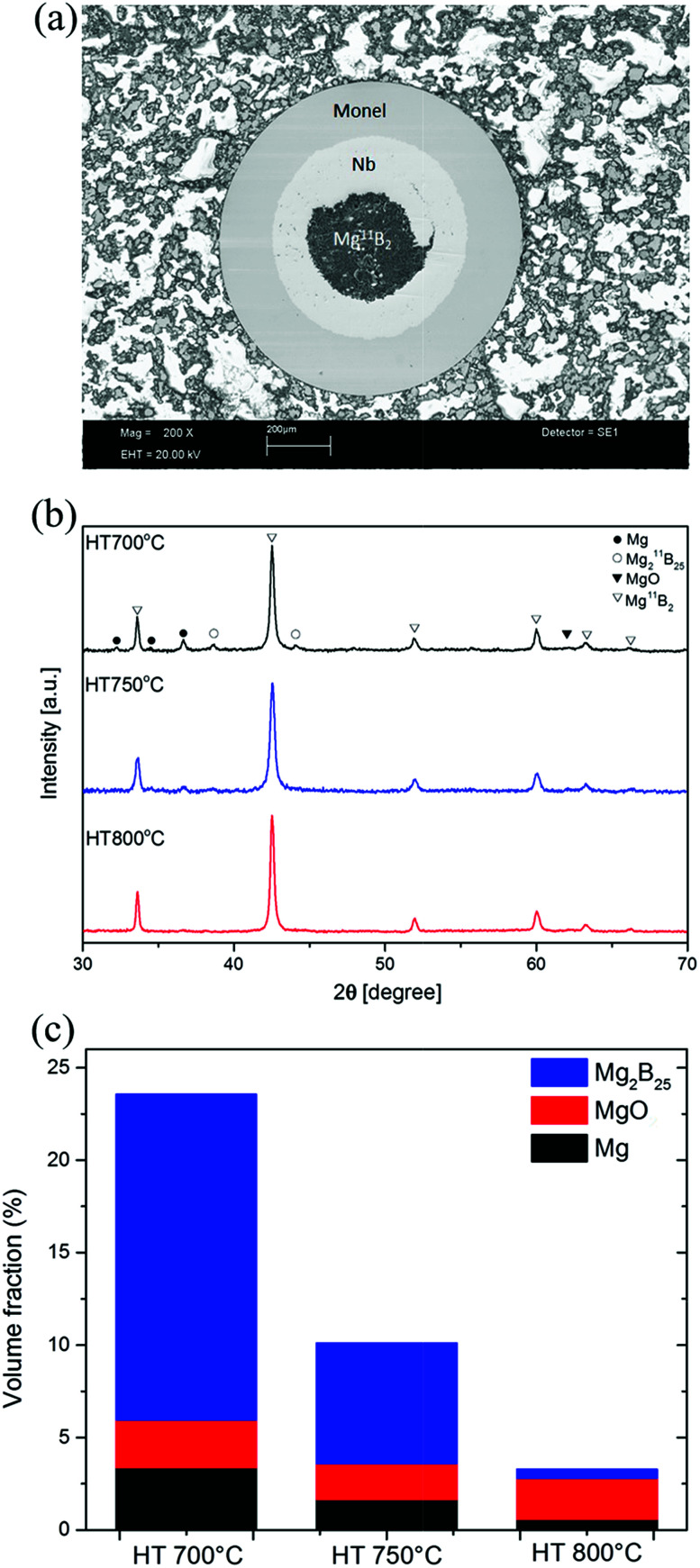
(a) The cross-sectional microstructure is shown in an SEM image of the Monel, Nb, and Mg^11^B_2_; (b) XRD patterns of the interior material of the Mg^11^B_2_ wires (Monel and Nb barrier are removed) after heat-treatment at 700 °C, 750 °C, and 800 °C; (c) the volume fractions of boron-rich phase (Mg_2_^11^B_25_), Mg, and MgO for Mg^11^B_2_ produced under different heat-treatment conditions.

Fig. 2(a) shows that the cross-sectional microstructure of the wire consists of 49 vol% Monel, 28 vol% Nb, and 23 vol% Mg^11^B_2_. Fig. 2(b) presents the XRD patterns of the superconducting ceramic from the core of the Mg^11^B_2_ wires after sintering for one hour at 700 °C, 750 °C, and 800 °C. While the major peaks are indexed as Mg^11^B_2_ phase, unreacted Mg, MgO, and ^11^B-rich phase^47,48^ are present in the samples. Fig. 2(c) shows the volume fractions of the secondary phases as functions of the sintering temperature. In the wires sintered at 700 °C and 750 °C, there are certain amounts of retained ^11^B-rich phase and Mg phase, 10–20 vol%, sufficient to produce significant and measurable microstresses. Further increasing the heat-treatment to 800 °C diminished the volume fraction of the Mg and the ^11^B rich phase to 0.37% and 0.47%, respectively, resulting in the most fully reacted, most pure Mg^11^B_2_ superconductor.

**Fig. 3:**
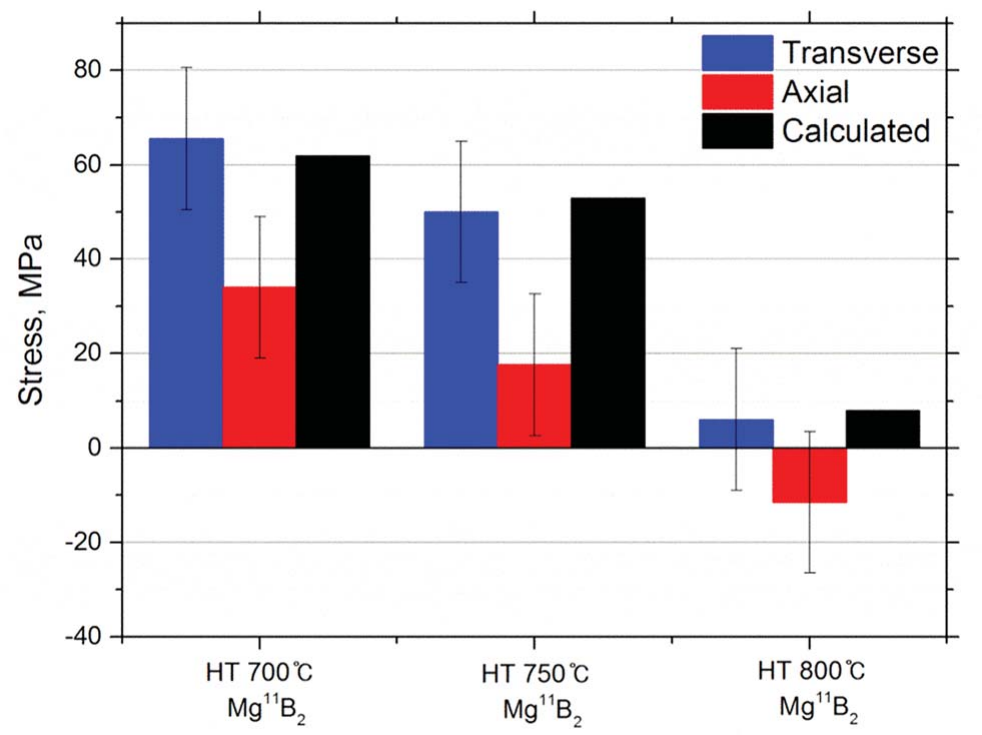
Residual stress in the Mg^11^B_2_ wires heat-treated at 700 °C, 750 °C, and 800 °C.

Fig. 3 shows the experimental results for the residual stress measurements of Mg^11^B_2_ wires in the transverse and the axial directions, with error bars showing the estimated uncertainty due to neutron counting statistics. The wires were characterised to have tensile stress of 66 ± 15 MPa (HT 700 °C), 50 ± 15 MPa (HT 750 °C), and 6 ± 15 MPa (HT 800 °C) for the transverse component, which had a tendency to decrease with increasing heat-treatment temperature to almost negligible in the HT 800 °C sample.

The approach of the stress analysis and interpretation is based on the decomposition of the total stress into micro- and macro-stress.^49^ In the given system it is deemed that both components are present due to the structure of the wire, there is an inner core, which is by itself is a composite material, and there is also a possible interaction between that core and our metal sheath.

The main contribution to the total stress was hydrostatic microstress (phase incompatibility stress) due to the interaction between the Mg^11^B_2_ matrix and the elastically harder ^11^B rich phase upon cooling down from the sintering temperature.^50^ It is generated due to the difference in the thermal expansion of the phases. This microstress is assumed fully thermally generated, since MgB_2_ phase is synthesised during the heat-treatment process, and isotropic, since all constituent phases are crystallographically isotropic. This experimental result was corroborated by evaluating thermally generated phase stresses using a micromechanical model of the isotropic particulate composite based on the Eshelby inclusion formalism.^51,52^ The calculations were made accordingly to the evaluated volume fractions (Fig. 2) of the constituents (Mg^11^B_2_ as the primary phase, plus unreacted ^11^B rich phase inclusions) and the thermal conditions for the composite formation in the Mg–B phase diagram.^53^ A good numerical agreement with the experimental results was achieved. (Fig. 3 combines the experimental and calculated results.) Thus, based on the XRD phase analysis results and the residual stress neutron measurements, it can be concluded that the higher heat-treatment temperature of 800 °C is required for the full reaction of the ^11^B rich and Mg phases to form Mg^11^B_2_, which ensures a low level of residual microstress.

In the axial direction, some compressive contribution to the total stress is present in addition to the hydrostatically-compressive microstress contribution discussed above, thus bringing the stress in the axial direction from tensile to less tensile, or even into compressive range, as in the 800 °C heat-treated wire. This effect can be explained by the interaction between the Monel-Nb sheath and the Mg^11^B_2_, and is due to thermally generated macrostress. Taking account of the differences in the coefficient of thermal expansion (CTE) of the sheath and wire interior (Δ*α*) and the temperature drop from the sintering temperature to room temperature (Δ*T*), the thermal strain mismatch Δ*ε* = Δ*T*·Δ*α* determines the sign and magnitude of the macrostress in the sheath and in the interior of the wire. Based on the CTEs of the constituents, *α* (Mg^11^B_2_) = 8.3 × 10^−6^/K,^54^*α* (Monel) = 14 × 10^−6^/K,^55^ and *α* (Nb) = 7.3 × 10^−6^/K,^56^ a compressive axial stress should be generated in Mg^11^B_2_, compensated by the tensile stress in the Monel sheath. In the wires sintered at 700 °C and 750 °C, with some amount of unreacted Mg phase and ^11^B rich phase, the same consideration is supposed to include Mg (*α* = 24.8 × 10^−6^/K)^57^ and ^11^B (*α* = 6 × 10^−6^/K)^58^ as well as microstructure features (*e.g.* possible pores and cracking). The resultant effect is highly sensitive to the conditions on the contact between the Monel tube and the Mg^11^B_2_ composite interior. Yet another explanation of this partial stress relaxation in the axial direction could be the presence of oriented cracks and pores arising from contraction during the sintering process and the pores originating from the Mg^11^B_2_ phase formation reaction in the heat-treatment procedure.

**Fig. 4:**
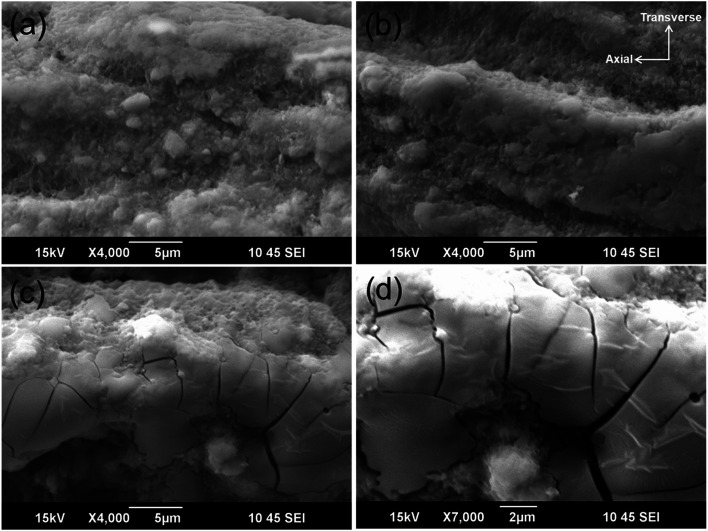
Low-vacuum SEM images of longitudinal sections of Mg^11^B_2_ wires heat-treated at (a) 700 °C, (b) 750 °C, and (c and d) 800 °C.

Fig. 4(a–d) shows the microstructure in the longitudinal direction of the Mg^11^B_2_ wires sintered at 700 °C, 750 °C, and 800 °C. These secondary electron image (SEI) observations indicate that aggregation occurs along with the presence of some small pores and microcracks in the 700 °C and 750 °C wires (Fig. 4(a and b)), while cracking and pores are more pronounced in the wire heat-treated at 800 °C (Fig. 4(c and d)). As the heat-treatment temperature increases, the aggregation of the Mg^11^B_2_ growth proceeds continuously while creating pores. As a result of the aggregation, Mg^11^B_2_ has a porous structure, and it can be easily damaged by thermal stress caused by the temperature drop from above 700 °C to room temperature.

Although the pores provide a precondition for the cracking-susceptible microstructure, the actual origin and mechanism of stress generation is twofold. First, due to the difference in CTE between the Monel/Nb sheath and the superconducting material, macrostress is generated, which in circumstances of porous microstructure leads to stress concentration. Second, due to the anisotropic thermal expansion of Mg^11^B_2_ (hexagonal crystal structure, *α*(*a*) = 5.4 × 10^−6^/K, *α*(*c*) = 11.4 × 10^−6^/K (ref. 59)), when the grains are randomly oriented, microstresses can also be generated. Although the overall average volume of these stresses is zero, the localised stresses can reach very large values, up to ∼1 GPa accordingly to our estimates. Thus, through these thermal mechanisms, very high magnitude and locally concentrated stress fields are generated, leading to microcrack formation conditions. The exact morphology, phase composition, and other details of the microstructure play roles in the actual stress state of the superconducting material. Thus, the more porous structure of the 800 °C sample makes it more cracking-prone than the lower temperature samples (700 °C and 750 °C) with more homogeneous structures. Also, while in less pure samples (700 °C and 750 °C), the local stress/strain fields can be accommodated by the plastically soft metallic Mg phase, this mechanism is substantially suppressed in the most pure (800 °C) sample, and thus, cracks are more easily formed in the 800 °C sample. Therefore, due to these two mechanisms, the most significant cracks on the scale of several microns are formed in the 800 °C sample.

Furthermore, the brittle fracturing leads to extensive cracking in the Mg^11^B_2_ structure, as seen in Fig. 4(c and d), resulting in a harmful effect on the transport *J*_c_ properties in Mg^11^B_2_ wire.^60,61^ In fact, it was previously reported in our research results that the wire sintered at 800 °C did not show a transport *J*_c_, even though the sample was fully reacted with a high Mg^11^B_2_ superconducting phase fraction.^62^ On the other hand, the wire sintered at 750 °C has a superior transport critical current density, *J*_c_ = 2 × 10^4^ A cm^−2^ at 4.2 K and 5 T compared with the multifilament wire manufactured by the National Institute for Fusion Science (NIFS).^62^ Therefore, based on the above discussion, the cracks and the pores have a detrimental influence on *J*_c_ through fracturing of the inter-grain connections, while, at the same time, these defects act as stress relief factors in the Mg^11^B_2_ wire sintered at 800 °C.

The superconducting transition temperature (*T*_c_) was observed at a temperature of 36.5 K and 36.9 K, for the samples sintered at 700 °C and 800 °C, respectively.^62^ Compared to the reported results, *e.g.* 39.2 K in,^63^ the lower *T*_c_ in the present samples is most likely due to the presence and complex interactions of different types of MgB_2_ lattice defects, such as lattice strains/stresses, poor crystallinity, the presence of point defects and defects with higher dimensions, and issues with chemical purity and phase purity (*e.g.* the presence of small amounts of MgO).^64^ The exact role of each factor might be difficult to address, however due to the intertwined nature of these mechanisms.^65^

## Conclusion

Due to its relevance to the superconducting properties, the stress state of Mg^11^B_2_ wires sintered (heat-treated) at different temperatures was investigated using neutron diffraction. We found that the stress in Mg^11^B_2_ is due to two contributions: one is the thermally generated hydrostatic microstress most clearly manifested in the transverse direction; the other is the contribution of the thermally generated macrostress, which has a uniaxial nature due to the “wire sheath-interior” interaction with its effects in the axial direction and/or possibly some contribution to stress relaxation due to oriented microcracking. We also found that, as the sintering temperature increases to 800 °C, it leads to the formation and growth of cracks in the superconducting ceramic as well as presence of some pores. These defects are, most likely, not related to the thermally generated stress, in so far as it is the lowest in the 800 °C sintered sample, but initiated during the sintering process itself and most likely involving the phase transformation mechanism. The extended cracking negatively affects the superconducting properties in the Mg^11^B_2_ wire, to the point of total loss of the superconductivity, even though the Mg^11^B_2_ superconducting ceramic sintered at 800 °C is the most pure and would be expected to have better properties because the high sintering temperature gives rise to stress relief in the Mg^11^B_2_ wire. In other words, the noticeable relaxation of the residual stresses in the axial and the transverse directions implies that the poor transport *J*_c_ value is caused by insufficient grain connection in the Mg^11^B_2_ wire. Overall, the micromechanical and structural features of the Mg^11^B_2_-based wires are essential for their performance, and neutron diffraction seems to be an appropriate analytical tool for assessment of the residual stress state as well as the crystallographic anisotropy (texture).

## Author contributions

H. J. and W. Q. prepared the samples, V. A. A., A. N. and Y. B. characterized the samples, D. G., J. K. and Y. Y. contributed to discussions on the obtained data, and V. L. and M. S. A. H. organized the manuscript.

## Conflicts of interest

The authors declare no competing financial interests.

## Supplementary Material
